# Intra-articular vs. systemic administration of etanercept in antigen-induced arthritis in the temporomandibular joint. Part II: mandibular growth

**DOI:** 10.1186/1546-0096-7-6

**Published:** 2009-02-06

**Authors:** Peter Stoustrup, Kasper D Kristensen, Annelise Küseler, Thomas K Pedersen, John Gelineck, Troels Herlin

**Affiliations:** 1 Department of Orthodontics, Faculty of Health Sciences, University of Aarhus, Vennelyst Boulevard 9, 8000 Aarhus C, Denmark; 2 Department of Radiology, Aarhus University Hospital, Nørrebrogade 44, 8000 Aarhus C, Denmark; 3 Department of Pediatrics, Aarhus University Hospital Skejby, Brendstrupgaardvej 100, 8200 Aarhus N, Denmark

## Abstract

**Background:**

Temporomandibular joint (TMJ) arthritis in children causes alterations in the craniomandibular growth. Resultant abnormalities include; condylar erosions, a posterior mandibular rotation pattern, micrognathia, malocclusion with an anterior open bite, altered joint and muscular function occasionally associated with pain. These alterations may be prevented by early aggressive anti-inflammatory intervention. Previously, we have shown that intra-articular (IA) corticosteroid reduces TMJ inflammation but causes additional mandibular growth inhibition in young rabbits. Local blockage of TNF-α may be an alternative treatment approach against TMJ involvement in juvenile idiopathic arthritis (JIA). We evaluated the anti-inflammatory effect of IA etanercept compared to subcutaneous etanercept in antigen-induced TMJ-arthritis in young rabbits in terms of mandibular growth. This article (Part II) presents the data and discussion on the effects on facial growth. In Part I the anti-inflammatory effects of systemic and IA etanercept administration are discussed.

**Methods:**

Arthritis was induced and maintained in the TMJs of 10-week old pre-sensitized rabbits (n = 42) by four repeated IA TMJ injections with ovalbumin, over a 12-week period. One group was treated weekly with systemic etanercept (0.8 mg/kg) (n = 14), another group (n = 14) received IA etanercept (0.1 mg/kg) bilaterally one week after induction of arthritis and one group (n = 14) served as an untreated arthritis group receiving IA TMJ saline injections. Head computerized tomographic scans were done before arthritis was induced and at the end of the study. Three small tantalum implants were inserted into the mandible, serving as stable landmarks for the super-impositions. Nineteen variables were evaluated in a mandibular growth analysis for inter-group differences. All data was evaluated blindedly. ANOVA and T-tests were applied for statistical evaluation using p < 0.05 as significance level.

**Results:**

Significant larger mandibular growth disturbances were observed in the group receiving IA saline injections compared with the systemic etanercept group. The most pronounced unfavourable posterior mandibular rotation pattern was observed in the group receiving IA saline injections.

**Conclusion:**

Intervention with systemic etanercept monotherapy equivalent to the recommended human dose allows a mandibular growth towards an original morphology in experimental TMJ arthritis. Systemic administrations of etanercept are superior to IA TMJ administration of etanercept in maintaining mandibular vertical growth.

## Background

Temporomandibular joint (TMJ) arthritis in growing individuals severely affects the endochondral ossification in the condylar cartilage responsible for a substantial part of the mandibular growth [1,2]. The intracapsular location of this growth cartilage is a unique characteristic of the TMJ and it makes this joint vulnerable to inflammatory changes. It is evident that the pronounced mandibular growth deviations seen in juvenile idiopathic arthritis (JIA) patients is a consequence of TMJ arthritis [3-5]. Resultant abnormalities include; condylar deformities, reduced vertical mandibular ramus growth, a critical posterior clockwise mandibular rotation pattern and reduced muscular and TMJ function, occasionally associated with pain, and a secondary affection of the maxilla and soft tissue [3-6]. JIA patients with TMJ involvements are mainly treated with orthopaedic procedures and corrective surgery after mandibular growth disturbances have occurred [7,8]. These treatment modalities are long of duration, comprehensive and require excellent patient cooperation. We hypothesize that early aggressive intervention against TMJ arthritis aimed against the inflammatory process *per se *would be advantageous and allow mandibular growth towards an original morphology. Beneficial symptomatic and functional improvements of intra-articular (IA) corticosteroid injections for the treatment of TMJ arthritis in children with JIA have been described [9-11]. However, these studies do not address the potential negative long-term effects of IA TMJ corticosteroid injections on condylar cartilage and craniofacial growth. This calls for attention since long-term side-effects on mandibular growth may outweigh the beneficial short-term effects. Previously, we have published data to support that, despite of significant inflammatory reduction, IA TMJ corticosteroid injections seem to have unfavourable effects on the mandibular growth in experimental studies [12,13]. As always, it requires caution and careful consideration when applying the findings in an experimental TMJ arthritis model to humans but our previous findings warrant the search for alternative local anti-inflammatory treatment procedures.

TNF-α plays an important role in the pathogenesis of JIA, and high levels of TNF-α are associated with a negative effect on the endochondral ossification [14-17]. Additionally, in rheumatoid arthritis (RA) TMJ pain and tissue destruction are associated with elevated synovial levels of TNF-α [18,19]. In polyarticular JIA not responding to standard DMARD therapy with methotrexate, the efficacy and safety of etanercept given subcutaneously have been well documented [20]. In RA patients IA etanercept has shown a good response in terms of symptomatic relief [21,22]. No previous study has addressed the topic of mandibular growth and anti-TNF-α therapy against TMJ arthritis.

In the present study we investigated the effect of systemic and IA administration of etanercept on mandibular growth in experimental antigen-induced TMJ arthritis in rabbits. We hypothesized that the anti-inflammatory treatment with TNF-α blockers would allow a mandibular growth towards an original morphology in antigen-induced TMJ arthritis compared to an IA saline treated group. The anti-inflammatory effects of the etanercept interventions are described in part I: Histological Effects [23].

## Methods

**Figure 1:**
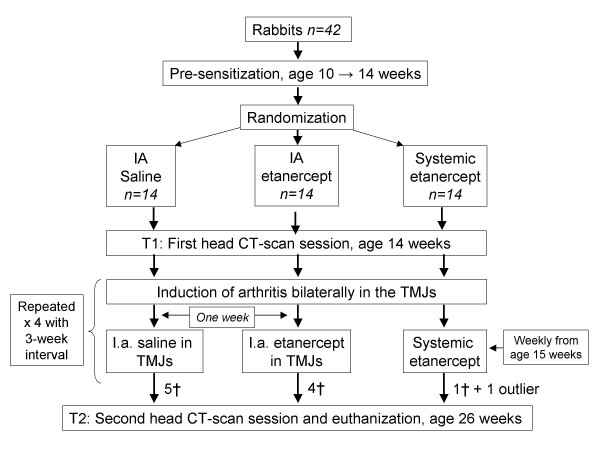
**Flow chart illustrating the study design**.

Ten-week old female New Zealand white rabbits (n = 42) (*Oryctolagus cuniculus*) were housed at the animal facilities of the University of Aarhus, Denmark, with free access to water and food. Animal welfare was monitored by daily evaluation of food and water intake. Prior to arrival the animals were randomly divided into three groups by block randomisation; a) IA TMJ saline group, n = 14 (0.1 ml pr. joint), b) IA TMJ etanercept group, n = 14 (0.1 mg/kg pr. joint), c) systemic etanercept group, n = 14 (0.8 mg/kg subcutanously). All animals had TMJ arthritis induced as described in Part I [23] (figure 1).

Prior to T1 all animals had three 1.0 × 0.33 mm tantalum implants inserted in the mandible serving as stable landmarks used in the mandibular growth analysis [1,24]. One implant was inserted close to the mandibular symphysis and another in the molar region in both sides of the mandible. Two sets of full head CT scans (Phillips/Mx8000 IDT 16) were taken at T1 and at T2 (age 26 weeks); resulting in a growth period of 12 weeks. All animals were sacrificed at age 26 weeks. Implant operation, TMJ injections and euthanization were carried out under general anaesthesia and all procedures were approved by the Danish Ethical Committee for animal welfare. IA injections and surgical procedures were carried out in a blinded fashion by trained specialists.

### Growth variables

**Figure 2:**
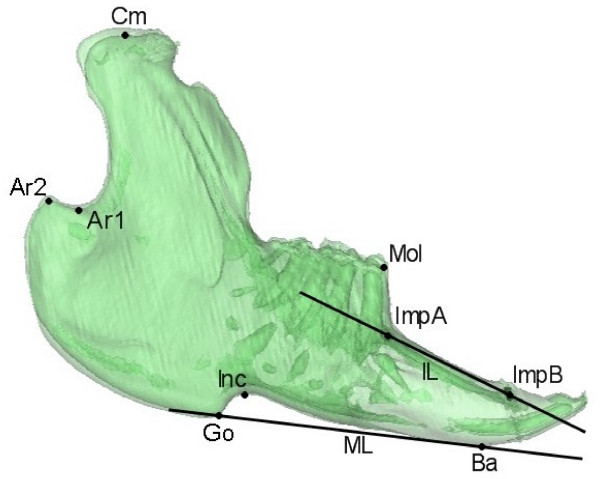
**Anatomical landmarks**. **Cm **(Condylar midpoint), **Ar1 **(Articulare 1), **Ar2 **(Articulare 2), **Go **(Gonion), **Ba **(Basion point), **Inc **(Incisal point), **Mol **(Molar point). The implant points were located on the right side: **ImpA **(Implant A) and **ImpB **(Implant B). The following two lines were defined: **ML **(Mandibular line) from Go to Ba, defined at the right side; **IL **(Implant line) going through ImpA-ImpB. For specific definitions of the anatomical landmarks see Stoustrup *et al*. 2008 [12].

**Figure 3:**
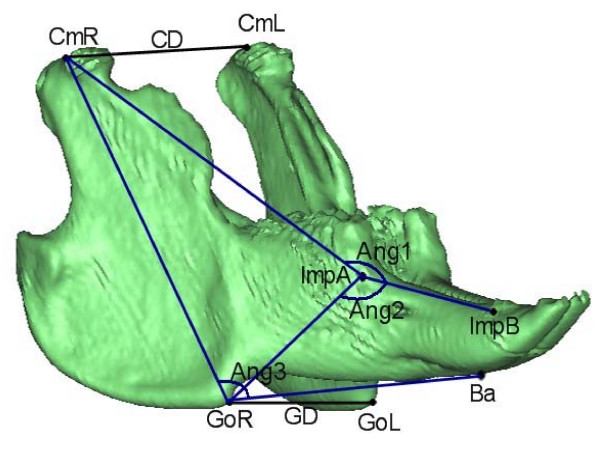
**Angles and transverse variables measured**. **Angle1**: Cm-ImpA-ImpB; **Angle2**: Go-ImpA-ImpB; **Angle3**: Cm-Go-Ba; **Condylar distance **(CD): Cm_right_-Cm_left_; **Gonial distance **(GD): Go_right_-Go_left_.

**Figure 4:**
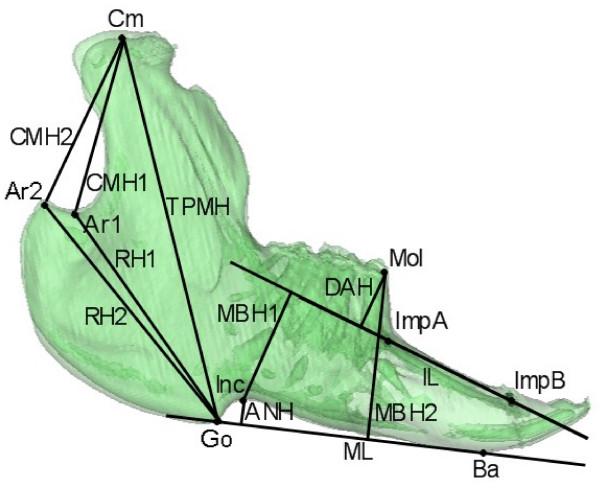
**Vertical variables measured**. **Total posterior mandible height **(TPMH): Cm-Go; **Collum mandibular height 1 **(CMH1): Cm-Ar1; **Collum mandibular height 2 **(CMH2): Cm-Ar2; **Ramus height 1 **(RH1): Ar1-Go; **Ramus height 2 **(RH2): Ar2-Go; **Mandibular body height 1 **(MBH1) Inc ⊥ IL; **Mandibular body height 2 **(MBH2): Mol ⊥ ML; **Dentoalveolar height **(DAH): Mol ⊥ IL; **Height of angular notch **(ANH): Inc ⊥ ML.

**Figure 5:**
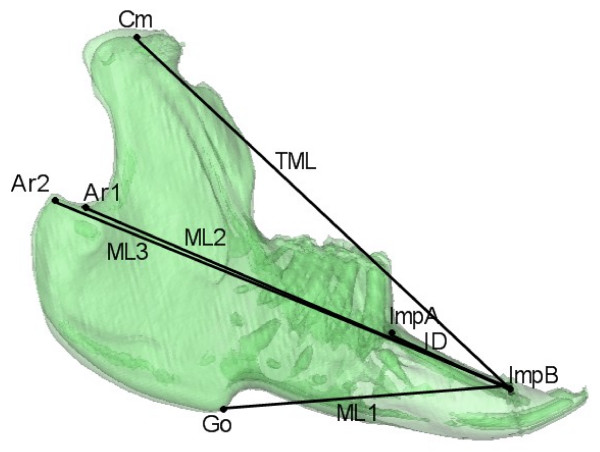
**Sagittal variables measured**. **Mandibular length 1 **(ML1) Go-ImpB; **Mandibular length 2 **(ML2) Ar1-ImpB; **Mandibular length 3 **(ML3) Ar2-ImpB; **Total mandibular length **(TML) Cm-ImpB; **Implant distance **(ID): ImpA-ImpB. Figures from Stoustrup *et al*. 2008 [12].

Evaluation of inter-group growth differences between T1 and T2 were evaluated by a mandibular growth analysis based on CT scans. Figure 2 shows the anatomical landmarks and implants used for definition of the variables evaluated. Figures 3, 4 and 5 show the 19 variables evaluated at T1 and T2 used in the analysis of mandibular morphology changes in each animal. Variables describing vertical and sagittal changes were obtained from the right side of the animals.

The scanned images were digitised by one author (PS) using the image evaluation software program Mimics (Mimics 11, Materialise, Leuven, Belgium) and all images were assessed in a blinded fashion before group assignment was revealed. Only statistical analyses prespecified in the investigation protocol were performed. The data were statistically processed using the software program Stata (Intercooled Stata 8.2, StataCorp, Texas, USA).

### Statistics

All continuing variables were evaluated for normal distribution. At T1, absolute inter-group differences for each of the nineteen variables were evaluated by one-way ANOVA-tests. Sagittal, vertical and transverse intra-group growth between T1 and T2 were evaluated by paired t-tests. In each of the nineteen variables differences in the relative inter-group growth between T1 and T2 were evaluated using one-way ANOVA-tests. Student's t-tests were applied as post-ANOVA tests in variables when significance was found in the ANOVA-tests. The necessity for a Bonferroni correction for avoidance of mass significance in the post-ANOVA inter-group Student's t-tests was discussed. However, to avoid type-2 errors due to the correlation between the nineteen variables evaluated in each animal, this correction was found too strong and not implemented in the statistical analysis. In a previous study, acceptable intra-observer variance and inter-scan accuracy were demonstrated using the same measuring methods, equipment and observer as in this study [13]. Power calculations were performed at T2 in order to evaluate the validity of our findings in relation to the number of animals completing the study. The parameters for the calculations were 2α = 0.05 and β = 20. Discussed below are only findings sufficiently powered. The level of significance used was p < 0.05.

## Results

Thirty-two animals completed the study. The distribution of the completers at T2 were; nine from the IA saline group, ten from the IA etanercept group and thirteen from the systemic etanercept group. One animal from the systemic etanercept group was excluded from the final growth analysis due to abnormal histological TMJ findings in this animal [23].

At T1 mandibular maturation was identical for all three study groups. This was confirmed by no absolute inter-group differences in any of the nineteen cephalometric variables at T1 (Table 1). Significant sagittal, vertical and transverse intra-group growth in almost all of the three groups was observed during the 12 weeks of growth (p < 0.05). An exception for this was the growth of the dentoalveolar height (DAH) in the IA etanercept group which did not demonstrate growth between T1 and T2. This is explained by the small growth increment and the large variation in the growth of this variable. The implant A to implant B distances did not change between T1 and T2. This documents the stable properties of the implants inserted. Between T1 and T2 angle 1 did not open significantly in any of the groups; Angle 2 closed significantly only in the IA saline group and Angle 3, signifying the angle between the ramus and the horizontal plane of the mandible, opened significantly in all three groups.

**Table 1:** Absolute intra-group differences between T1 (week 14) and T2 (week 26) together with mean relative difference for each of the variables in each group evaluated.

Variable	Group	Absolute mean T1 value	Absolute mean T2 value	Absolute mean difference T2-T1	Relative mean difference from T1 to T2 (%)	Standard deviation (%)	Statistical significance for growth between T1 and T2
Angle 1	A	155.3	156.6	1.3	0.84	1.7	n.s.
	B	155.8	156.4	0.6	0.4	2.9	n.s.
	C	153.9	154.1	0.2	0.1	1.6	n.s.
Angle 2	A	136.1	133.8	-2.3	-1.7	2.2	*
	B	136.2	135.1	-1.1	-0.7	5.2	n.s.
	C	138.7	137.4	-1.3	-1	2.2	n.s.
Angle 3	A	108.3	109.3	1	0.9	1.2	*
	B	107.3	108.6	1.3	1.3	1.5	*
	C	108.3	109.6	1.3	1.2	1.3	*

Sagittal (mm)							

TML	A	61.5	66.6	5.1	8.3	2.1	*
*(Cm-ImpB)*	B	60.7	65.8	5.1	8.4	1.9	*
	C	60.8	66.2	5.4	8.8	1.8	*
ML1	A	36.2	38.1	1.9	5	2.4	*
*(Go-ImpB)*	B	36.5	38.2	1.7	4.6	2.5	*
	C	35.9	37.6	2	5	2.1	*
ML2	A	55.6	59.7	4.1	7.3	1.7	*
*(Ar1-ImpB)*	B	55.3	59.0	3.7	7.2	2.2	*
	C	54.8	58.7	3.9	7.2	2.6	*
ML3	A	58.6	63.3	4.7	8	1.8	*
*(Ar2-ImpB)*	B	58.6	63.1	4.5	7.7	0.9	*
	C	58.3	62.8	4.5	7.7	1.4	*
ID	A	15.1	15.5	0.4	3	6.2	n.s.
*(impA-impB)*	B	14.8	15.1	0.3	2.7	5.8	n.s.
	C	14.1	14.0	-0.1	-1.3	8.1	n.s.

Vertical (mm)							

TPMH	A	44.2	48.8	4.6	10.4	1.5	*
*(CmR-GoR)*	B	43.3	47.9	4.6	10.8	1.9	*
	C	43.5	49.0	5.5	12.6	1.7	*
CMH1	A	21.4	23.7	2.3	10.7	1.1	*
*(CmR-Ar1)*	B	21.1	23.2	2.1	9.9	3.5	*
	C	21.3	23.6	2.3	10.8	2.6	*
CMH2	A	22.3	24.3	2	8.9	2.0	*
*(CmR-Ar2)*	B	22.2	24.3	2.1	9.4	2.9	*
	C	22.1	24.4	3.3	10.0	2.3	*
RH1	A	27.4	30.3	2.9	10.7	2.5	*
*(Ar1-GoR)*	B	26.8	29.7	2.9	11.0	2.3	*
	C	26.8	29.9	3.1	11.6	3.5	*
RH2	A	29.5	32.9	3.4	11.9	3.4	*
*(Ar2-GoR)*	B	28.9	32.5	3.6	12.3	2.5	*
	C	29.2	32.9	3.7	12.7	2.7	*
MBH1	A	12.5	13.8	1.3	10.4	13.9	*
*(IncR⊥IL)*	B	12.7	13.4	0.7	6.91	16.3	*
	C	12.3	13.6	1.3	12.1	12.4	*
MBH2	A	18.3	20.5	2.2	12.2	3.7	*
*(MolR⊥IL)*	B	17.9	19.8	1.9	11.1	2.9	*
	C	17.7	20.2	2.5	14.1	3.9	*
DAH	A	7.5	8.2	0.7	8.9	10.6	*
*(MolR⊥ML)*	B	7.3	7.9	0.6	8.8	14.4	n.s.
	C	7.7	8.7	1	12.6	8.4	*
ANH	A	2.9	3.4	0.5	17.2	18.2	*
*(IncR⊥ML)*	B	2.9	3.4	0.5	17.2	26.9	*
	C	2.6	2.9	0.3	11.5	35.3	*

Transverse (mm)							

GD	A	28.1	29.6	1.5	5.5	2.8	*
*(GoR-GoL)*	B	29.0	30.1	1.1	3.9	2.0	*
	C	28.4	29.9	1.5	5.5	2.5	*
CD	A	33.4	34.7	1.3	4.3	2.4	*
*(CmR-CmL)*	B	32.5	33.9	1.4	4.5	2.2	*
	C	33.2	34.4	1.2	3.7	1.3	*

*P < 0.05

Angles (degrees)

Groups: A) placebo, B) IA etanercept, C) systemic etanercept. See figures 3, 4 and 5 for a description of the variables.

**Figure 6:**
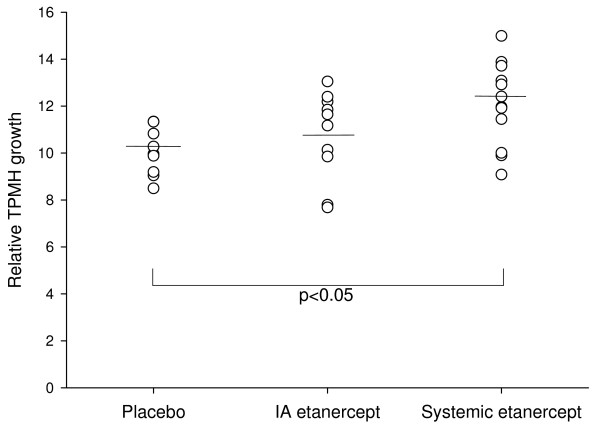
**The relative growth of the total posterior mandibular height (TPMH)**. The growth of this variable was significantly larger (p < 0.05) in the systemic etanercept group (TPMH 12.6% [CI-95: 11.0–13.1]) compared with the IA saline group (TPMH 10.4% [CI-95: 9.2–11.5]). A non-significant trend (p < 0.1) towards a difference in the relative TPMH growth was observed between the systemic etanercept group (TPMH 12.6% [CI-95: 11.0–13.1]) and the IA etanercept group (TPMH 10.8% [CI-95: 9.2–12.2]).

Evaluation of relative inter-group differences in mandibular growth during the 12 weeks of growth observed we found no significant differences in terms of angular, sagittal and transverse mandibular maturation and growth. In the vertical dimension a significant relative inter-group difference in mandibular growth was observed in the total posterior mandibular height (TPMH) (p < 0.05). Post-ANOVA tests showed that the relative growth of the total posterior mandibular height (TPMH) was significantly larger in the systemic etanercept group (p < 0.05), but not for the IA etanercept group, compared with the IA saline group of animals (figure 6). A non-significant trend (p < 0.1) towards a difference in the relative growth of the TPMH was observed between the systemic etanercept group and the IA etanercept group. In variables such as the mandibular body height 1 (MBH1) and the antegonial notching height (ANH) large inter-group differences in the relative growth were observed but due to the combination of large variations and a small growth in these variables no significant differences were observed.

## Discussion

**Figure 7:**
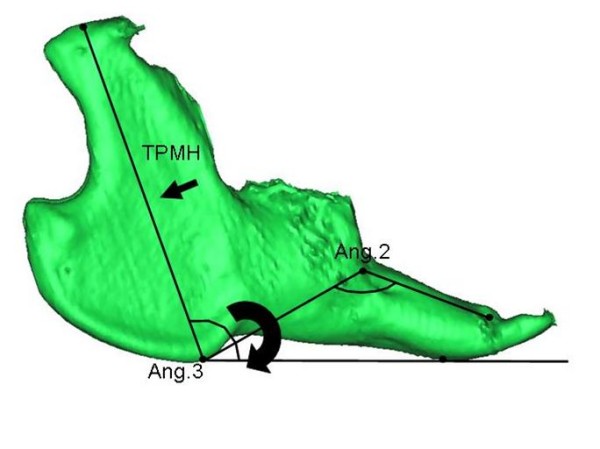
**The findings of this study**. Intervention with systemic etanercept monotherapy equivalent to the recommended human dose allows mandibular growth towards an original morphology by significantly improved growth of the total posterior mandibular height (see TPMH variable at small arrow). Systemic etanercept (0.8 mg/kg weekly) administration is superior to IA TMJ etanercept administration at times of inflammatory flares in maintaining mandibular vertical growth. All three groups demonstrated an unfavourable clockwise posterior mandibular rotation pattern, illustrated by an opening of Angle 3 (large arrow). The most pronounced posterior mandibular rotation was observed in the IA saline group in which a closing of Angle 2 also occurred.

In this study we have demonstrated that subcutaneous etanercept administration, in a weekly dose of 0.8 mg/kg, allows a mandibular growth towards an original morphology. Additionally, we found that systemic etanercept is superior to IA etanercept administration (0.1 mg/kg at times of inflammatory flares) in supporting mandibular vertical growth. In terms of mandibular rotational growth, all three experimental groups demonstrated an unfavourable posterior clockwise mandibular rotation pattern illustrated by an opening of the mandibular angle (Angle 3) (figure 7). However, most critical posterior rotation pattern was observed in the IA saline group in which a closing of Angle 2 occurred additionally. Reduced vertical mandibular growth and mandibular posterior rotation are two of the most pronounced and critical clinical features in JIA patients with inflammatory TMJ changes [3,5]. Both of these features are signs of very unfavourable mandibular growth [1,24].

The findings in this study indicate that the mandibular growth is most efficiently supported by a weekly systemic anti-inflammatory and immunomodulatory therapy compared to a local anti-inflammatory therapy at times of inflammatory flares. However, there are several possible reasons for the lack of effect of IA etanercept in this study that need further attention. It is not known how long IA administered etanercept stays in the joint before being absorbed and entering circulation: etanercept has an elimination half life between 70.8 and 96.4 hours [25]. Despite of an expected higher concentration of TNF-α inhibitor within the TMJs, achieved by IA injections compared to systemic etanercept administration, the duration of IA injected etanercept seems to have an insufficient clinical effect on the TMJ arthritis to result in beneficial long-term results on mandibular growth. The concentration of the IA etanercept injected could therefore have been insufficient. The dose of the systemically administered etanercept (0.8 mg/kg subcutaneously) used in this study is in agreement with the recommended weekly dose of etanercept in humans [20]. This dose is not equivalent to the dose of IA etanercept administered and this may explane our findings. At the beginning of this study the IA etanercept concentration used (0.1 mg/kg) was decided based on the sparse information published on IA etanercept in letters and case reports. After initiating our study, Bliddal *et al*. described good symptomatic and functional results by the use of IA etanercept injections in RA patients (wrist, knee or elbow) with injections of 0.3 mg/kg etanercept (25 mg/joint) [22].

Aspects to consider in future studies are whether to give IA injections more frequently than at times of flares, increase the IA injected etanercept concentration, use another TNF-α inhibitor with a prolonged elimination, or to see if the effect of IA etanercept injections would have been improved when supported by another anti-inflammatory or immunomodulatory systemically administered therapy. Recently, repeated IA infliximab (Remicade^®^, Centocor) injections in the TMJ in RA patients have been described with good results [26].

In the present study, the IA saline group and IA etanercept group animals received more TMJ injections per animal than the systemic etanercept group animals. Histologically, we have shown that IA TMJ saline injections *per se *do not result in any inflammatory or destructive changes in the joint [12]. Therefore, the fact that the number of TMJ injections per joint *per se *being lower in the systemic etanercept group does not seem to be of crucial importance for the influence on mandibular growth.

It requires special caution and careful consideration when applying findings in experimental studies to humans. In this study we used etanercept (a humanized TNF-α receptor fusion protein) for the treatment of inflammation in rabbits. However, etanercept has a documented effect on rabbit inflammation as described in Part I of this study [23]. Additionally, non-pathological rabbit mandibular growth resembles non-pathological human mandibular growth [27]. The experimental arthritis model used in this study is well described [28]. It shows similar mandibular growth deviations in rabbits compared to the pathologically mandibular growth deviations seen in JIA children with inflammatory TMJ changes [29].

In rabbits approximately 90% of the overall mandibular growth is achieved before the sixteenth week and has almost ceased at age 26 weeks [30]. Yet, launching this study at an earlier animal age was not possible due to Danish legislation on animal welfare. Systemic presensitization with antigen was initiated in age 10 weeks and the first IA TMJ antigen injections were introduced at age 14 weeks. In rabbits the greatest mandibular growth acceleration is seen in the early postnatal period and reduces at age 14 weeks [30], so obviously it would have been preferable to initiate this trial in younger animals. In this study, the significant inter-group differences in mandibular vertical growth occur within the final 10–15% of the overall mandibular growth. Therefore, we hypothesize that greater inter-group differences in several mandibular growth variables could have been revealed if the trial was initiated in younger animals during the growth acceleration because of an increased mandibular growth potential left. This may explain the lack of differences on angular, sagittal and transverse measurements. Ten animals did not complete the full length of the study and it is questionable whether TMJ arthritis induction could have been initiated at an earlier animal age without extensive loss of animals. All animals lost in the experimental phase were lost due to anaphylactic shock right after the IA TMJ antigen injections. The majority of lost animals died during the first two antigen challenge procedures.

The orthopaedic treatment of JIA patients with TMJ inflammation is long of duration, comprehensive and requires good patient cooperation. Often the clinical signs of TMJ arthritis are vague and the disorder is often overlooked [6]. Early aggressive intervention against TMJ involvement aimed against the inflammatory process *per se *could allow mandibular growth towards an original morphology. Symptomatic and functional improvements of IA corticosteroid injections for the treatment of TMJ arthritis in children have been described [9,11]. However, previously we have published data supporting that, despite of significant inflammatory reduction, these injections have a highly unfavourable effect on the mandibular growth in experimental studies [12,13]. Therefore, searches for alternative local anti-inflammatory treatments are warranted in the future. This study suggests that early intervention with systemic etanercept monotherapy weekly is beneficial and allows growth towards an original morphology. It could be of great clinical interest to consider the effect of systemic etanercept as a compliment to an orthopaedic mandibular correction with orthodontic splint therapy in the intervention against early mandibular growth alteration in JIA patients. Another interesting clinical aspect is if a synergistic effect on mandibular growth can be achieved with a combination of methotrexate and systemic anti-TNF-α administration. Data have documented that monotherapy with methotrexate reduces TMJ destruction and growth alterations and craniofacial dysmorphology in polyarticular JIA children with TMJ arthritis [31]. In RA patients a synergistic good therapeutic effect on structural joint damage has been described when methotrexate and systemic anti-TNF-α therapy have been combined [32].

## Conclusion

Treatment of experimental TMJ arthritis with systemic etanercept monotherapy weekly, equivalent to the recommended human dose, allows mandibular growth towards an original morphology which is unlike our previous findings with IA corticosteroids for the treatment of experimental TMJ arthritis. Weekly systemic etanercept administrations are superior to IA TMJ etanercept administration, at times of antigen induced inflammatory flares, in maintaining mandibular vertical growth.

## Abbreviations

JIA: Juvenile Idiopathic Arthritis; TMJ: Temporomandibular Joint; IA: Intra-articular; TNF: Tumor Necrosis Factor; CT-scan: Computerized Tomography scans; RA: Rheumatoid Arthritis.

## Competing interests

The authors declare that they have no competing interests.

## Authors' contributions

PS, KDK, AK, TKP and TH designed and conducted the experiment and decided upon the context of the growth analysis. JG was responsible for CT-scan sessions and other aspects of these sessions. PS conducted all the measurements on the CT-scans and was responsible for the statistical analysis. PS drafted the initial manuscript. All authors have made significant contribution to the manuscript regarding content, interpretation and read and approved the final manuscript.
